# RETRACTION: LncRNA AATBC Regulates Pinin to Promote Metastasis in Nasopharyngeal Carcinoma

**DOI:** 10.1002/1878-0261.70314

**Published:** 2026-08-07

## Abstract

**RETRACTION:** T. Tang, L. Yang, Y. Cao, M. Wang, S. Zhang, Z. Gong, F. Xiong, Y. He, Y. Zhou, Q. Liao, B. Xiang, M. Zhou, C. Guo, X. Li, Y. Li, W. Xiong, G. Li, and Z. Zeng, “LncRNA AATBC Regulates Pinin to Promote Metastasis in Nasopharyngeal Carcinoma,” *Molecular Oncology* 14, no. 9 (2020): 2251‐2270, https://doi.org/10.1002/1878‐0261.12703.

The above article, published online on 4 May 2020 in Wiley Online Library (https://onlinelibrary.wiley.com/), has been retracted by agreement between the journal Editor‐in‐Chief, Kevin M. Ryan; the Federation of European Biochemical Societies; and John Wiley & Sons Ltd. The retraction has been agreed due to concerns raised by third parties. Specifically, image elements in Figures 2D and 2G were found to have been previously published by the author group in different scientific contexts. Furthermore, the GAPDH blots derived from the CNE2 cells for the “NC” and “siPNN” groups in Figure 4B, and for the “NC” and “1237‐3p” groups in Figure 5C were found duplicated. In addition, overlapping regions were identified between the panels depicting the “NC” and “siAATBC + PNN” groups within the CNE2 lane.

While the authors acknowledged the errors and supplied raw data in support of their response, the editors deemed the clarification insufficient to address their concerns. The authors attributed the mistakes to similarities between the affected images, raising concerns over the accuracy of the data presented in the remaining body of the article. Furthermore, further review determined that the vast majority of the cited references were self‐citations, many of which were found to be irrelevant or only marginally relevant to the context at hand, resulting in several statements lacking adequate support from the literature. Accordingly, the article has been retracted, as the editors have lost confidence in the reliability of the results and conclusions presented. The authors have been informed of the retraction decision, but were not available for final confirmation.

**RETRACTION:** T. Tang, L. Yang, Y. Cao, M. Wang, S. Zhang, Z. Gong, F. Xiong, Y. He, Y. Zhou, Q. Liao, B. Xiang, M. Zhou, C. Guo, X. Li, Y. Li, W. Xiong, G. Li, and Z. Zeng, “LncRNA AATBC Regulates Pinin to Promote Metastasis in Nasopharyngeal Carcinoma,” *Molecular Oncology* 14, no. 9 (2020): 2251‐2270, https://doi.org/10.1002/1878‐0261.12703.

The above article, published online on 4 May 2020 in Wiley Online Library (https://onlinelibrary.wiley.com/), has been retracted by agreement between the journal Editor‐in‐Chief, Kevin M. Ryan; the Federation of European Biochemical Societies; and John Wiley & Sons Ltd. The retraction has been agreed due to concerns raised by third parties. Specifically, image elements in Figures 2D and 2G were found to have been previously published by the author group in different scientific contexts. Furthermore, the GAPDH blots derived from the CNE2 cells for the “NC” and “siPNN” groups in Figure 4B, and for the “NC” and “1237‐3p” groups in Figure 5C were found duplicated. In addition, overlapping regions were identified between the panels depicting the “NC” and “siAATBC + PNN” groups within the CNE2 lane.

While the authors acknowledged the errors and supplied raw data in support of their response, the editors deemed the clarification insufficient to address their concerns. The authors attributed the mistakes to similarities between the affected images, raising concerns over the accuracy of the data presented in the remaining body of the article. Furthermore, further review determined that the vast majority of the cited references were self‐citations, many of which were found to be irrelevant or only marginally relevant to the context at hand, resulting in several statements lacking adequate support from the literature. Accordingly, the article has been retracted, as the editors have lost confidence in the reliability of the results and conclusions presented. The authors have been informed of the retraction decision, but were not available for final confirmation.

